# Breast cancer risk during oral contraceptive use in women with high polygenic risk

**DOI:** 10.1186/s13058-025-02177-5

**Published:** 2025-12-01

**Authors:** Christina Chatsatourian, Valeria Lo Faro, Torgny Karlsson, Fatemeh Hadizadeh, Åsa Johansson

**Affiliations:** Department of Immunology, Genetics and Pathology, SciLifeLab, Uppsala University, Uppsala, Sweden

**Keywords:** Oral contraceptives, Breast cancer, Polygenic risk score, Genetic susceptibility, Epidemiology, UK biobank

## Abstract

**Background:**

Oral contraceptive (OC) use is widespread globally. Despite their significant benefits, concerns persist about a potential rise in breast cancer risk linked to their use. Genetic predisposition also influences breast cancer risk; however, its interaction with OC use remains inconclusive. This study aims to explore the association between OC use and breast cancer risk in women with varying genetic predispositions to breast cancer, as measured by polygenic risk scores (PRS).

**Method:**

A total of 257,185 white female participants from the UK Biobank were included. Time-varying Cox regression was used to estimate hazard ratios (HRs) with 95% confidence intervals (CIs) to examine the association between OC and invasive breast cancer events, stratified by PRS. Age was used as the primary time scale, and analyses were adjusted for year of birth, Townsend Deprivation Index, body mass index, smoking status, age at menarche, menopausal status, family history of breast cancer, parity, hormone replacement therapy use, history of hysterectomy, as well as genetic principal components.

**Results:**

Current use of OC was associated with an increased risk of breast cancer, with a HR of 1.21 (95% CI: 1.03–1.41). In contrast, previous use showed no association (HR = 0.99, 95% CI: 0.92–1.05). Genetic risk, as measured by the PRS, was strongly associated with breast cancer risk (*P* < 0.001). Individuals in the highest PRS decile had approximately three times higher risk compared to those in the mid deciles. Importantly, for the association between current OC use and breast cancer risk, a statistically significant trend was observed across both PRS deciles (*P* = 0.04) and tertiles (*P* = 0.05), with decreasing HRs as genetic risk increased. Specifically, the HR for current OC use was 1.43 (95% CI: 1.02–2.01) in the lowest PRS tertile, 1.14 (95% CI: 0.89–1.45) in the middle tertile, and 0.96 (95% CI: 0.80–1.14) in the highest tertile.

**Conclusion:**

Both OC use and a high PRS increase the risk of breast cancer. There is a trend toward a decreased relative risk associated with OC use among those with higher genetic predisposition. Therefore, there is no evidence to suggest that women with a high genetic risk for breast cancer are more adversely affected by OC use.

**Supplementary Information:**

The online version contains supplementary material available at 10.1186/s13058-025-02177-5.

## Background

Since their introduction in the early 1960s, oral contraceptives (OC) have been widely used and in 2024, the number of users of modern contraceptive methods globally was estimated to be 871 million [1]. Despite their widespread use, concerns about their potential carcinogenic risks have been raised since their introduction. It is well established that the risk of breast cancer is increased during OC use. For example, one of the largest studies conducted to date, a collaborative re-analysis of individual data from 53,297 women with breast cancer and 100,239 controls across 54 epidemiological studies, presents robust findings indicating that current users of OC have an increased risk (relative risk of 1.24) of breast cancer [2]. Similarly, more recent studies provide strong evidence that the use of hormonal contraceptives is associated with a higher risk (relative risk of 1.20 or hazard ratio of 1.24) of breast cancer compared to never users [3, 4].

Breast cancer remains a significant public health challenge worldwide, representing the most common malignancy among women. Genetic predisposition plays a significant role in the development of breast cancer, with up to 27% of cases exhibiting strong hereditary components [5, 6]. Around 5–10% of all breast cancer cases are linked to high-impact germline mutations in breast cancer susceptibility genes. Among these, up to 30% are attributed to pathogenic mutations in *BRCA1* and *BRCA2*, while mutations in other genes, including *TP53*, *PTEN*, *CHEK2*, *STK11*, and *PALB2*, account for a smaller proportion [7]. The few studies investigating the difference in magnitude of the association between OC use and breast cancer risk depending on genetic predisposition have primarily focused on carriers of the *BRCA1* and *BRCA2* mutations [8–10]. These studies indicate that the association between OC use and breast cancer risk may be weaker among women with a high genetic susceptibility.

However, not all women with an increased genetic risk of developing breast cancer are necessarily carriers of high-penetrance mutations. Over 300 Single Nucleotide Polymorphisms (SNPs) associated with breast cancer risk have been identified [11]. These SNPs are more prevalent in the general population than the pathogenic variants in high-risk genes. While each SNP individually contributes a small increase in breast cancer risk, their combined effect might account for up to 45% of the familial breast cancer risk [12]. Therefore, polygenic risk scores (PRS) have been suggested as a complementary tool for accurate breast cancer genetic risk prediction, as they evaluate the cumulative impact of multiple susceptibility variants identified through genome-wide association studies (GWAS) [13].

Even though both OC use and genetic susceptibility are known to be associated with increased risk of breast cancer, yet few studies have explored how genetic factors interact with OC use to influence breast cancer risk. This study aims to fill this gap by incorporating PRS to assess the impact of genetic predisposition on modulating the association between OC use and breast cancer risk.

## Methods

### Study cohort

This study utilized data from the UK Biobank (UKB). Between 2006 and 2010, over 500,000 men and women, aged 40 to 69 years, were recruited from the general population through 22 assessment centers across the United Kingdom [14, 15]. Participants provided extensive health-related data through questionnaires, interviews, physical examinations, and biological sample collection, with nearly all participants undergoing genotyping. Their medical history continues to be tracked through national registers, including hospital records, cancer registries, and death registries. This comprehensive dataset allows for in-depth analysis of health outcomes and risk factors. The UKB includes data from 273,375 women, of whom 257,185 self-identified as White (Irish, British, or other white backgrounds), who were included in this study.

### Study design, outcome and exposure variables

In the UKB, information on OC use was collected retrospectively through self-report interviews at the time of recruitment. Participants were asked about their previous use of OC via four specific questions: whether they had ever used OCs, the age at which they started using them, the age at which they stopped, and whether they were current users of oral contraceptives. Data on invasive breast cancer incidence and age at diagnosis were obtained from hospital admission records, cancer registries, and death registries [16]. A detailed description of the data fields and coding used to identify breast cancer cases and age at diagnosis is provided in Supplementary Table S1.

**Fig. 1:**
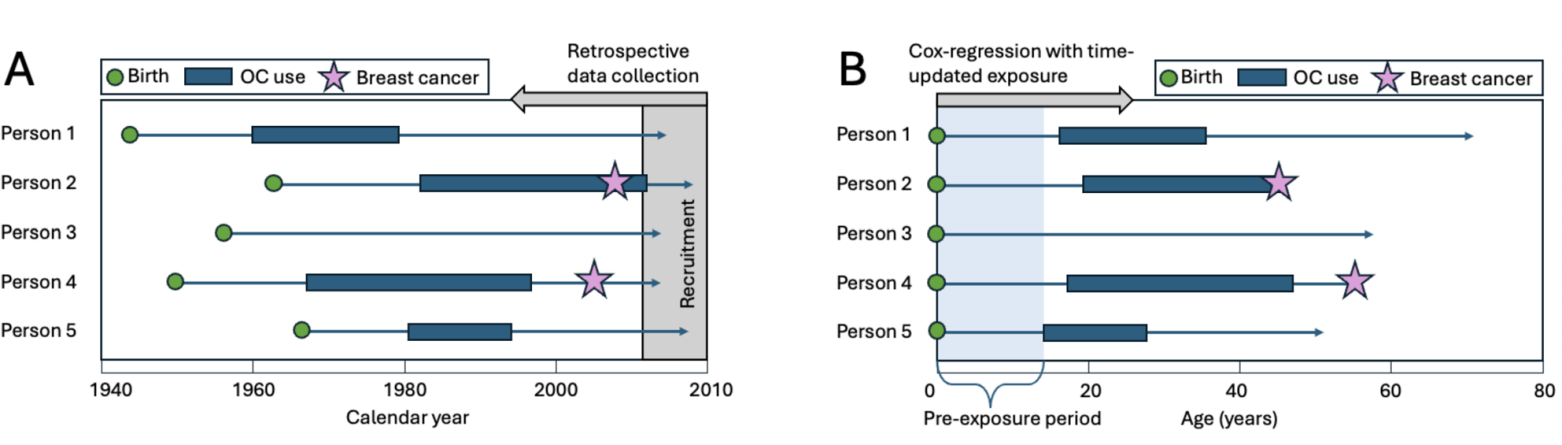
Data collection and study design. (**A**) Timeline for five hypothetical individuals with different birth years and varying periods of oral contraceptive (OC) use. All data used in this study were collected retrospectively at the time of recruitment to the UK Biobank, between 2006 and 2010. (**B**) In the statistical analyses, age is used as the primary time scale: all participants are aligned by age, and birth year is included as a covariate. Women are followed starting from birth (age = 0). However, the period before any participant had been exposed (the pre-exposure period highlighted in B) does not contribute to the exposure effect estimate. Follow-up continues until the age at recruitment or until a breast cancer event, whichever occurs first

We compiled a data frame that included the age at which changes in OC exposures occurred (i.e., when women started and stopped using OCs), whether participants had received a breast cancer diagnosis by the end of the follow-up, and if so, the age at which they were first diagnosed with breast cancer. Since the PRS is constant across a lifetime, it was included as a time-fixed variable. Age was used as the primary time scale [17]. In Cox models, the primary time scale (in this case, age) is fully and non-linearly adjusted for. This is an inherent property of the model’s mathematical structure, which ensures that both linear and non-linear age-related confounding is naturally accounted for. This can be especially relevant for outcomes such as breast cancer, which increase sharply with age.

The data collection and study design are illustrated in Fig. 1. Briefly, with an exposure status updated over time, women are coded as non-users before initiating treatment, as current users during active use, and as previous users after discontinuation. Women who are never users are coded as non-users until the end of follow-up. In the Cox regression modelling, the start of follow-up was set at age 0. Since the first period represents pre-exposure time (Fig. 1), as defined as the time when no women in the study have yet started to use OC, this time-period does not contribute to the exposure estimate. Women were followed until the first occurrence of breast cancer diagnosis, death, or the end of study follow-up, whichever came first. End of follow-up was defined as the age at initial assessment for each participant, which was the time point when information on previous OC use and all covariates was collected. Follow-up was not extended beyond this point, primarily because data on OC use were unavailable thereafter, and only a minority of women (< 2%) were still current users at the time of assessment. Consequently, the analyses were based on prevalent (at recruitment) breast cancer cases.

Two main analyses were conducted to analyse breast cancer risk in relation to OC use. First, comparing ever versus never users of OC, where women were coded as never-users initially and re-coded as ever-users once initiating OC use and until the end of follow-up. Second, comparing current versus never users as well as previous vs. never users of OC, where never-users were recoded as current users when they initiated OC use and until two years after discontinuation and then as previous users until the end of the follow-up. The coding of users as current until two years after discontinuation was done to mitigate protopathic bias, which could arise if some women stopped using OC after the appearance of early manifestations of breast cancer, while the diagnosis is registered after discontinuation [18]. For more information on the data sources used to extract the exposure variables, see Supplementary Table S2.

### Polygenic risk scores (PRS)

The PRS for breast cancer were precomputed by Genomics PLC under UKB project 9659 and provided through the UKB (data field 26220). Briefly, the standard PRS used in our study was constructed using GWAS summary statistics from cohorts that did not overlap with UK Biobank as described previously [19] and validated and benchmarked in relation to other PRSs [20].

Participants with missing data on their PRS were excluded from all analyses involving PRS. Participants were stratified into deciles based on their PRS, with decile 1 representing the lowest genetic risk and decile 10 the highest. The association between PRS deciles and breast cancer risk were assessed using logistic regression, with the medium PRS decile as the reference group. We also included the PRS in the time-dependent Cox regression models to assess a potential interaction between genetic susceptibility and OC use on breast cancer risk. The PRS was modelled both as a continuous variable and as a categorical variable, by stratifying participants into tertiles and deciles, thereby defining low-, medium-, and high genetic risk groups.

### Covariates

To account for potential confounders in the association between OC use and breast cancer risk, we included several covariates based on established risk factors. Reproductive variables such as age at menarche and menopausal status, parity, hormone replacement therapy (HRT) use, and hysterectomy were included due to their known linkage to breast cancer development [21, 22]. Anthropometric factors, such as body mass index (BMI), were included due to their well-documented association with breast cancer risk, with effects varying by menopausal status [23]. Socioeconomic status was adjusted for using the Townsend Deprivation Index (TDI), as socioeconomic disparities have been linked to differences in breast cancer diagnosis and outcomes [24]. Year of birth was included to adjust for cohort effects and temporal trends in reproductive behaviours and risk factors. Also, family history of breast cancer, and the first 10 genetic principal components were included as covariates.

Covariates for which temporal information was available were coded as time-varying. These included menopausal status, hysterectomy, HRT, and smoking, where data was collected retrospectively during the initial assessment (2006–2010). Several covariates are, by definition, fixed and do not vary over the follow-up period, including year of birth and age at menarche. For covariates known to vary over time, but for which no specific age or date was provided in the dataset, the value recorded at the initial assessment was used as a proxy throughout the study follow-up.

These fixed variables included TDI, BMI and, in the never-ever analyses, also number of live births. Participants who responded with “do not know,” “prefer not to answer,” or did not provide an age/year for the time-varying covariates were excluded from all analyses involving the respective covariates. Information on covariates and potential confounders was obtained from data collected during the initial assessment center visit (Supplementary Table S2), and the number of participants with missing values for each covariate is provided in Table 1. Smoking status was also considered due to evidence suggesting a potential positive association with breast cancer risk [25]. However, approximately 30,000 participants were excluded due to missing smoking status data. As a result, all models in the main analysis were run without adjusting for smoking status to maximize the sample size. However, sensitivity analyses were conducted to evaluate whether adjusting for smoking status would significantly affect the results (Supplementary Tables S3 – S4).

**Table 1:** Baseline characteristics of participants stratified by oral contraceptive use (OC)

Variable*	Entire cohort (*N* = 257,185)		*N* missing data	After filtering out participants with missing data (*N* = 190,332)		
	OC ever users	OC never users	N missing data (%)	OC ever users	OC never users	*P*-value**
Number of participants (%)	210,970 (82.0)	46,215 (18.0)	0	154,821 (81.3)	35,511 (18.7)	-
Breast cancer events, n (%)***	15,214 (7.21)	3,972 (8.59)	0	9,122 (5.89)	2,597 (7.31)	< 0.05
Positive family history of breast cancer, n (%)***	2,784 (1.32)	598 (1.29)	0	1,640 (0.78)	450 (0.97)	< 0.05
Year of birth, median (Q1–Q3)	1952 (1946–1959)	1945 (1942–1951)	0	1953 (1947–1960)	1946 (1942–1952)	< 0.05
Body mass index (kg/m2), median (Q1–Q3)	25.98 (23.36–29.53)	26.49 (23.69–30.11)	1,041 (0.4)	25.76 (23.21–29.24)	26.32 (23.57–29.89)	< 0.05
Age at recruitment, median (Q1–Q3)	56 (49–62)	63 (57–66)	0	55 (48–61)	63 (56–66)	< 0.05
Age at first breast cancer diagnosis, median (Q1–Q3)	56.3 (49.7–64)	60.2 (52.4–67.7)	0	55.4 (49.2–63.1)	59.6 (51.8–67.3)	< 0.05
Age at oral contraceptive initiation, median (Q1–Q3)	20 (18–24)	-	7,477 (2.91)	20 (18–24)	-	-
Age at oral contraceptive discontinuation, median (Q1–Q3)	30 (26–36)	-	25,760 (10.02)	31 (26–37)	-	-
Duration of oral contraceptive use in years, median (Q1–Q3)	9.0 (4–15)	-	-	10.9 (4–16)	-	-
Age at menarche, median (Q1–Q3)	13 (12–14)	13 (12–14)	7,305 (2.84)	13 (12–14)	13 (12–14)	0.21
Age at menopause, median (Q1–Q3)****	50 (48–53)	50 (48–53)	109,681 (42.65)	50 (48–53)	50 (48–53)	< 0.05
Number of children, median (Q1–Q3)	2 (1–2)	2 (0–3)	201 (0.08)	2 (1–2)	2 (0–3)	< 0.05
History of hysterectomy, n (%)	13,738 (5.34)	4,908 (1.90)	30,088 (11.7)	10,363 (6.6)	4,086 (11.5)	< 0.05
Townsend deprivation index (TDI), median (Q1–Q3)	-2.27 (-3.7–0.17)	-2.2 ( -3.62-0.39)	300 (0.12)	-2.30 (-3.71-0.11)	-2.20 (-3.64-0.37)	< 0.05
Current or previous smoker, n (%)	90,176 (35)	15,789 (6.13)	896 (0.35)	65,486 (42.29)	12,036 (33.89)	< 0.05
Polygenic risk score (PRS), median (Q1–Q3)	-0.14 (-0.81-0.54)	-0.13 (-0.82-0.55)	8,669 (3.37)	-0.14 (-0.82-0.54)	-0.14 (-0.82-0.55)	0.47

*Data are reported as number and percentage for categorical variables and median with interquartile range (Q1–Q3) for continuous variables.

**Significant p-values (< 0.05) suggest differences between OC users and non-users across various characteristics, estimated using the Chi-square test (for categorical variables) or Wilcoxon ranksum test (for quantitative values).

*** Only prevalent cases (diagnosed prior the recruitment) are included.

****In the analyses, menopause was coded as a time-varying covariate and women who had not reached menopause at end of follow-up (and thereby had missing information on age at menopause) were coded as pre-menopausal throughout the study.

### Statistical analysis modelling

**Fig. 2:**
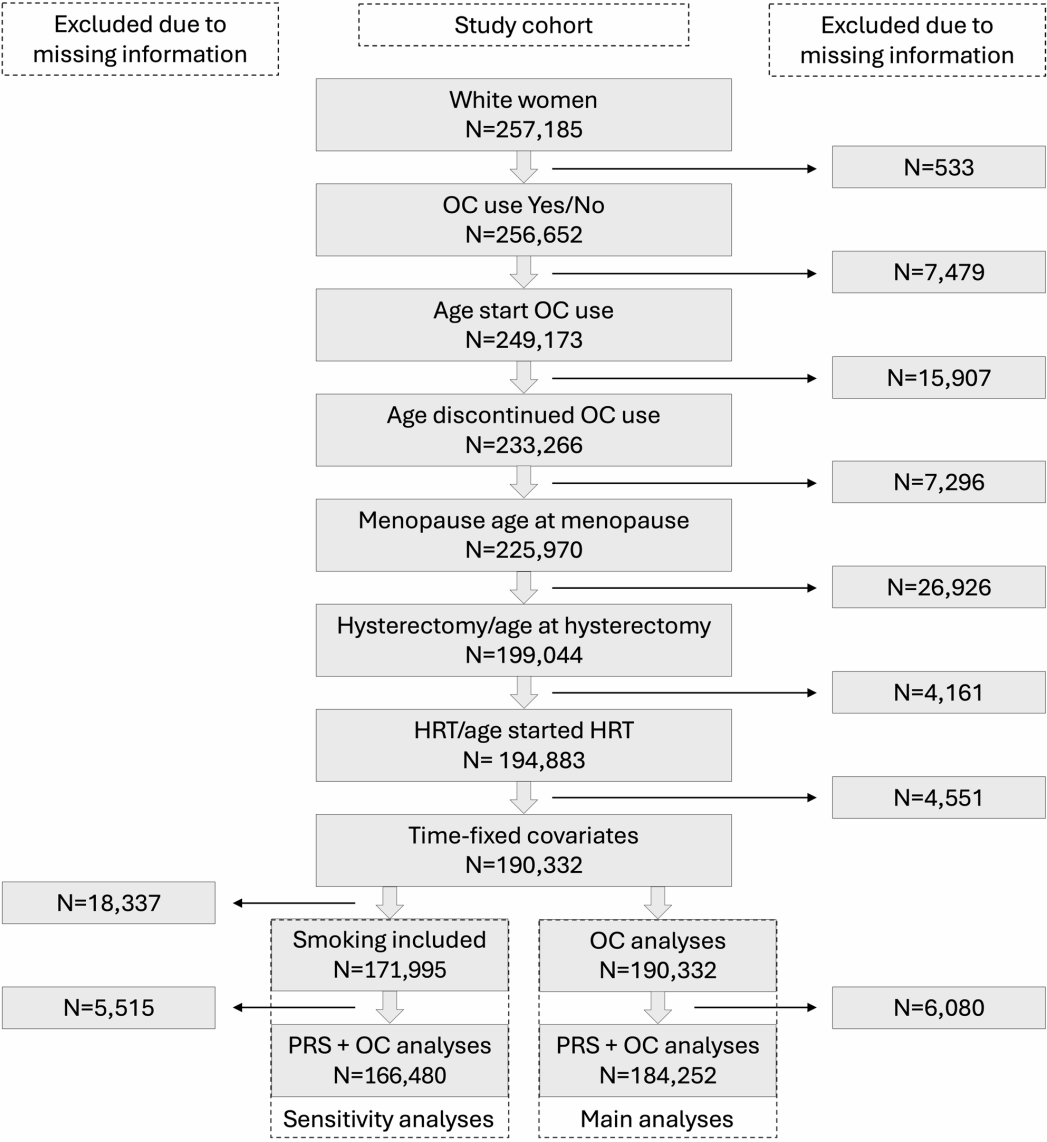
Flow chart of the main study cohort and information on exclusions due to missing information in the primary and sensitivity analyses where smoking was included as a covariate. For time-varying covariates (age at menopause, hysterectomy, and hormone replacement therapy [HRT]), women who did not provide exposure status for these variables, or who reported exposure but did not specify age at initiation, were excluded. Time-fixed covariates included number of children, age at menarche, body mass index (BMI), and the Townsend Deprivation Index (TDI). For further details on missing data, see Table 1

All participants with incomplete data required for statistical analyses were excluded (Fig. 2). OC use was modelled as a time-varying exposure using the counting process method [26–28], implemented with the tmerge() function in the R package *survival*. In this approach, individuals transitioned from an unexposed state to an exposed state at the time of their first OC use. The time-varying representation ensures accurate classification of the “event-free” period prior to OC initiation as unexposed time [29]. The reference group consisted of never-users, defined as women who had never used OC and those who had not yet initiated using OC during follow-up but contributed person-years before starting OC use. In the Cox regression models, the OC exposure was modelled as a factor variable. Hence, the hazard ratio (HR) for the first occurrence of breast cancer was estimated for ever users versus never users of OC, for current vs. never users, and for previous vs never users along with the 95% confidence interval (CI). To assess whether the HR associated with OC use varied across levels of genetic risk, we performed a trend test. A linear regression model was fitted with log(HR) as the dependent variable and PRS decile or tertile as the independent variable. The significance of the slope was used to evaluate the presence of a linear trend across genetic risk strata. All analyses were performed using the R version 4.3.1 and all Cox regression models were fitted using the “coxph” function from the *survival* package.

## Results

Of the 257,185 women self-identified as white, many lacked information on contraceptive use or study covariates, resulting in a final study population of 190,332 women (Fig. 2).

The characteristics of the study participants are summarized in Table 1 with most women (81.3%) having used OC at some point during the follow-up. The number of breast cancer events was lower among OC users compared to never users (5.9% and 7.3% respectively; *p* < 0.05), which most likely is explained by the lower age at the end of follow-up (recruitment to UKB) and which also agrees with a lower age at first breast cancer. OC ever users also had a lower median BMI compared to never-users and a lower TDI. Among ever-users, the median age at OC initiation was 20 years, with a median discontinuation age of 31 years and a median duration of 10 years. Age at menarche and menopause did not differ significantly between groups (*p* > 0.05). OC users were more likely to have had a hysterectomy (*p* < 0.05). Smoking was more prevalent among OC users (*p* < 0.05), whereas PRS did not differ significantly between groups (*p* > 0.05).

### Association between oral contraceptive (OC) use and breast cancer risk

**Fig. 3:**
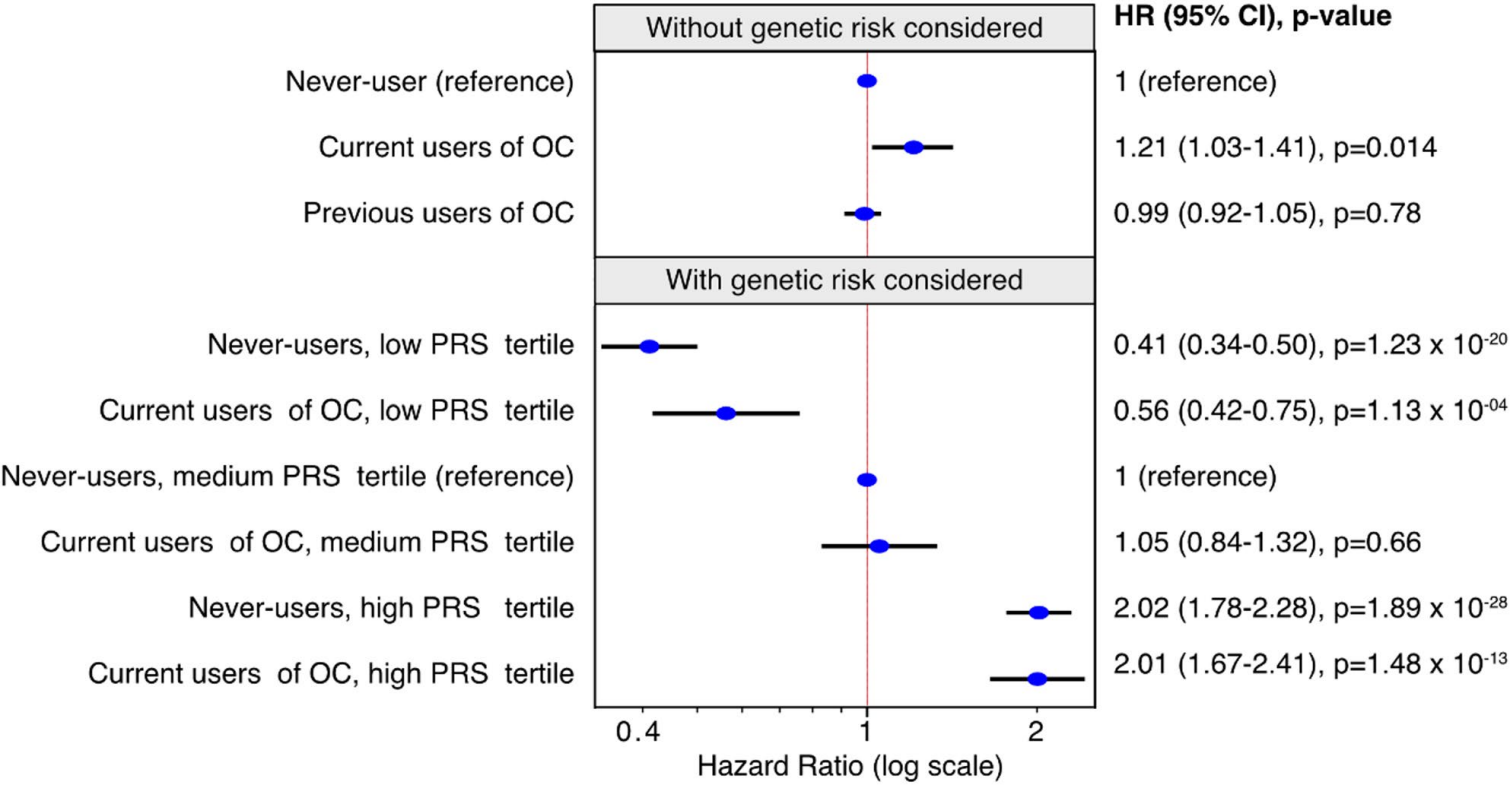
Hazard ratios (HR) for breast cancer associated with oral contraceptive (OC) use. In the upper panel, genetic risk was not considered, and a total of *N* = 190,332 women were followed, with a total of 2,394 women receiving a breast cancer diagnosis during the follow-up. OC use was coded as a time-varying exposure, and 81.2% of the women were current users at some point during the follow-up. A total of 2,133,453 person-years as never-users, 1,688,083 as during use, and 295,451 after use were accumulated. In the lower panel, participants were stratified depending on their genetic risk, with medium risk corresponding to the mid tertile of the polygenic risk score (PRS), and high genetic risk being the third tertile of the PRS (the 33.3% of the women with the highest PRS) and the low risk being the first tertile of the PRS. Hazard ratio (HR) and 95% confidence intervals (CI) for the respective group compared to the same reference (never users with medium genetic risk) are shown

We assessed the association between OC use and breast cancer risk (Fig. 3 upper panel). Individuals currently using OC had a significantly increased risk of breast cancer compared to those unexposed: HR = 1.21 (95% CI: 1.03–1.41), with 2,394 events observed during use. We did, however, not see any increased risk in the previous user group, HR = 0.99 (0.92–1.05).

When the quantitative PRS variable was added to the model with OC use as the exposure, the PRS was strongly associated with an increased breast cancer risk (Table 2), with an HR = 1.84 (1.75–1.93) per standard unit increase in PRS. In this model, the association between current OC use and breast cancer remained significant (HR = 1.23; 95% CI: 1.06–1.44), with a similar magnitude compared to the model without PRS (Table 2). The model including an interaction term (PRS*OC) indicated a small, but non-significant decrease in the HR (0.92; 0.82–1.04) for OC per standard unit increase in PRS (Table 2).

**Table 2:** Association between current oral contraceptive (OC) use, polygenic risk score (PRS), and their interaction in relation to breast cancer risk

Exposure	Without PRS***		With PRS**		With PRS x OC interaction term	
	HR (95% CI)*	P-value	HR (95% CI)*	P-value	HR (95% CI)*	P-value
Current OC use	1.21 (1.04–1.41)	0.012	1.23 (1.06–1.44)	0.006	1.28 (1.08–1.50)	0.003
PRS for breast cancer			1.84 (1.75–1.93)	< 0.001	1.87 (1.77–1.97)	< 0.001
PRS*OC (interaction term)					0.92 (0.82–1.04)	0.20

* Age was used as the primary time scale, and analyses were adjusted for year of birth, Townsend Deprivation Index, body mass index, smoking status, age at menarche and menopausal status, parity, family history of breast cancer, genetic principal components, hormone replacement therapy use, and history of hysterectomy.

** The PRS is analysed as a unstratified quantitative variable.

*** This analysis includes only women with available PRS data resulting in a very small difference in the CI and P-value compared to Fig. 3.

**Fig. 4:**
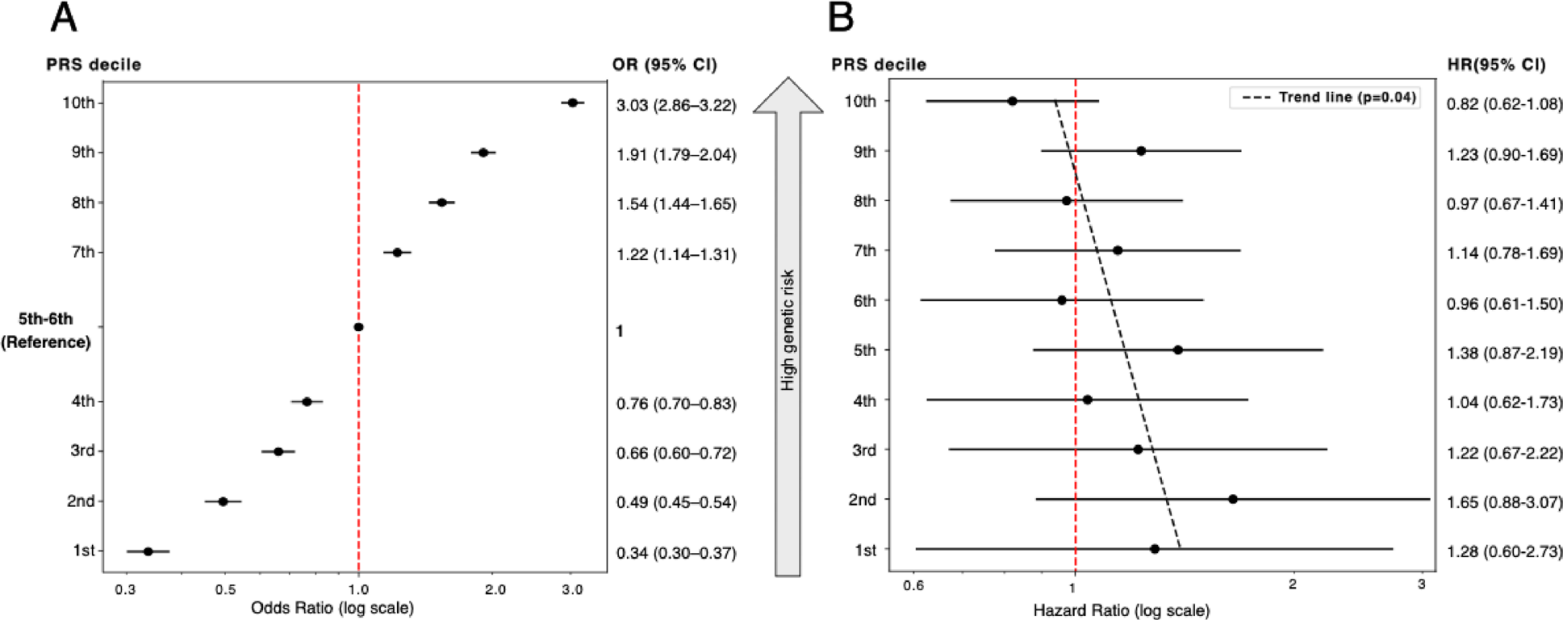
Odds ratios (OR) for breast cancer according to polygenic risk score (PRS) deciles and hazard ratios (HR) for breast cancer by current oral contraceptive (OC) use stratified by genetic risk. (**A**) ORs with 95% confidence intervals (CI) for developing invasive breast cancer during follow-up are shown for each PRS decile, using the medium genetic risk group (deciles 5–6) as the reference. (**B**) HRs for invasive breast cancer associated with current OC use across ten strata defined by PRS deciles with never users within each decile serving as the reference group. The dashed black line indicates the trend (*p* = 0.04), showing decreasing HRs for breast cancer with increasing genetic risk

Stratifying women into deciles of the PRS showed a dramatically increased risk in women in the highest decile compared to the middle deciles (Fig. 4A), with nearly a threefold higher risk. Conversely, women in the lowest PRS decile showed a similarly reduced risk compared to the middle deciles, indicating a clear gradient of genetic susceptibility across the PRS deciles. The association with OC use was further investigated when women were stratified into low, moderate and high genetic risk groups based on their PRS. Here, the reference group consisted of women unexposed to OC (never users) with medium genetic risk (mid-tertile of the PRS). The results indicated that among women with medium genetic risk, current OC use was not associated with a significantly increased risk of breast cancer (HR = 1.05, 95% CI: 0.84–1.32, *p* = 0.61) (Fig. 3). Among individuals in the high genetic risk group (top tertile of the PRS), both never users and current OC users had an increased risk of breast cancer of similar magnitude (HR = 2.02, 95% CI: 1.78–2.28 and HR = 2.02, 95% CI: 1.67–2.41, respectively). In the low genetic risk strata (the lowest tertile of the PRS), both current and never users showed a decreased risk (HR = 0.56, 95% CI: 0.42–0.75 and HR = 0.41, 95% CI: 0.34–0.50, respectively). Using the same stratification by PRS tertiles, we estimated the HR for breast cancer associated with current OC use within each tertile, using never users in the same stratum as the reference group (Table 3). Only the low genetic risk strata showed a statistically significant increased risk by OC use with HR of 1.43 (1.02–2.01) and 1.21 (1.00–1.46) in current and previous users. Seemingly, the HR for OC use tended to decrease with increasing genetic risk, which was consistent with the trend seen for the interaction term when the PRS was analysed as a quantitative variable (Table 2). To analyse this further, we further stratified genetic risk into 10 PRS deciles. Although no individual stratum reached statistical significance, a significant trend was observed (*p* = 0.04), with HRs for breast cancer associated with OC use decreasing across PRS deciles (Fig. 4B).

**Table 3:** Hazard ratios (HR) for breast cancer by oral contraceptive (OC) use stratified by genetic risk-

Genetic risk	OC user group	HR (95% CI)	*P*-value
Low (1st tertile of the PRS)	Never-users	1.00 (reference)	-
	Current users	1.43 (1.02–2.01)	0.040
	Previous users	1.21 (1.00–1.46)	0.046
Medium (2nd tertile of the PRS)	Never-users	1.00 (reference)	-
	Current users	1.14 (0.89–1.45)	0.295
	Previous users	0.93 (0.82–1.06)	0.276
High (3rd tertile of the PRS)	Never-users	1.00 (reference)	-
	Current users	0.96 (0.80–1.14)	0.630
	Previous users	0.97 (0.89–1.06)	0.507

HR = hazard ratio; CI = confidence interval; PRS = polygenic risk score

## Discussion

In this study, we conducted a comprehensive analysis of the association between OC use and breast cancer risk while accounting for polygenic risk of breast cancer. The HR of 1.21 for breast cancer associated with current OC use in our study is consistent with previous research reporting a 20–25% higher risk among OC users [2–4].

These patterns have been shown to be consistent across various populations, regardless of reproductive history, family history of breast cancer, or ethnic background. Our finding, that the increased breast cancer risk was observed during OC use but was less pronounced after discontinuation, agrees with previous studies [4] and supports the hypothesis that hormonal exposure from OC may shorten the latency period for breast cancer development, as suggested by previous studies [30, 31], rather than contributing to a sustained higher lifetime risk. However, whether women with a high genetic risk for breast cancer are more affected by the harmful effects of OC use has not previously been conclusively investigated. This knowledge is crucial for personalized risk prediction and contraceptive counselling.

The use of PRS for breast cancer risk prediction has been increasingly discussed during the last years [32–34]. We showed here that having a high PRS is associated with a much larger breast cancer risk compared to OC use. However, there was no evidence that OC use is associated with higher breast cancer risk in women with higher genetic predisposition; if anything, a significant trend suggested a weaker association in this group. Our results align with previous studies examining the relationship between OC use and breast cancer risk in *BRCA1* and *BRCA2* mutation carriers. Although several studies have been conducted, they have found no evidence that current or recent use of OC further elevates the risk of breast cancer among *BRCA1/2* mutation carriers [8–10]. It is also worth noting that although OC use appears to increase breast cancer risk slightly less, or not at all, in women with high genetic risk, these women are already at elevated baseline risk. Prescribing contraceptives to women with a high genetic risk should, nevertheless, be approached with caution, and the findings from this and previous studies [8–10] warrant further validation before informing any clinical recommendations. The UKB PRS used in this study was developed by Genomics PLC and shows a slightly higher hazard ratio compared with the most commonly used breast cancer PRS in clinical contexts, PRS313 [34]. While the overall patterns of association are expected to be similar for other widely used PRSs, the exact magnitudes of the observed associations may vary across datasets.

This study has several strengths and limitations. It benefits from a large sample size and a prospective cohort study design with age as the primary time-scale that minimizes the risk of confounding by age and we have adjusted for relevant covariates. Multiple analyses and models of oral contraceptive use were conducted, including an adjustment for genetic risk, providing a comprehensive evaluation of the association between oral contraceptive use and breast cancer risk. However, the study is limited by its focus on white women living in the UK, which may affect the generalizability of the findings to other ethnic groups [35]. Also, many of the covariates, as well as the exposure were self-reported, which may have introduced recall bias and led to missing data, primarily due to participants responding: “do not know.” Since the disease outcome was determined using national register data, it is unlikely that recall bias or missingness in the self-reported variables would be systematically associated with errors in the register-based outcome. Therefore, while recall bias and missing data may have reduced statistical power, it is unlikely to have inflated the estimated effect sizes. We also lack information on breast cancer subtypes. Additionally, and most importantly, the analysis did not evaluate different types of hormonal contraceptives. Most women in the UK Biobank are at an age where they have predominantly used second-generation combined (estrogen and progestin) oral contraceptive pills [30]. In the last decade, new types of contraceptives with different formulations and routes of administration have gained popularity, including progestin-only products, implants and intrauterine devices. However, no large-scale cohort currently exists that includes data on contemporary hormonal contraceptives, genetics, and sufficiently long follow-up periods.

## Conclusions

Given the widespread use of OC, understanding the effects of these medications is essential for shaping public health strategies and guidelines. Overall, our findings contribute to the growing body of evidence suggesting that any increase in breast cancer risk associated with current OC use in the general population may be transient. Importantly, the risk appears to decline after discontinuation, with little to no lasting impact on lifetime breast cancer risk once hormonal exposure has ended. However, individual risk factors, such as genetic predisposition, as assessed by the PRS, could refine the interpretation of these effects. A personalized approach, accounting for a woman’s unique genetic profile, family history, and other risk factors, is essential to guide informed choices regarding OC and improve clinical decision-making in breast cancer prevention.

Our results, together with previous research, suggest that OC use may not pose additional risk specifically for women with high genetic predisposition, but it could still represent a modifiable risk factor for those with elevated baseline risk. However, the several beneficial effects of hormonal contraceptives underscore the importance of not discouraging their use unnecessarily. Contraceptive agents play a crucial role in global health by enabling individuals to make informed reproductive choices, promoting family planning, and improving health outcomes. Access to modern contraceptives has contributed significantly to the prevention of unintended pregnancies, unsafe abortions, and maternal deaths. In addition, OC use has been associated with increased bone mineral density and a reduced risk of fractures later in life [36], as well as a significantly lower risk of ovarian and endometrial cancer [30, 31, 37, 38]. In relation to genetic risk, this is particularly important for ovarian cancer, given the overlap between several breast cancer susceptibility genes and those associated with an increased risk of ovarian cancer, one of the malignancies with the highest mortality rates, for which OC use has been identified as one of the most effective preventive factors [30, 38]. Therefore, additional studies exploring personalized risk profiles and the impact of OC use are urgently needed to refine risk assessments and improve clinical recommendations.

## Supplementary Information

Below is the link to the electronic supplementary material.

Supplementary Material 1

## Data Availability

The data analysed in this project are available from the UKB resource (https:/ukbiobank.ac.uk) and can be accessed via relevant application to the UKB. The code used in the analyses in this study is available on GitHub (https://github.com/AJResearchGroup).

## References

[1] Nations Department of Economic, Affairs U. S. DESA Policy Brief 172 - March 2025.

[2] Calle EE, Heath CW, Miracle-McMahill HL, Coates RJ, Liff JM, Franceschi S et al. Breast cancer and hormonal contraceptives: Collaborative reanalysis of individual data on 53 297 women with breast cancer and 100 239 women without breast cancer from 54 epidemiological studies. Vol. 347, Lancet. 1996. pp. 1713–27. 10.1016/s0140-6736(96)90806-5 8656904

[3] Hadizadeh F, Koteci A, Karlsson T, Ek WE, Johansson Å. Hormonal contraceptive formulations and breast cancer risk in adolescents and premenopausal women. JAMA Oncol. 2025. 10.1001/jamaoncol.2025.4480 PMC12576617 41165687

[4] Mørch LS, Skovlund CW, Hannaford PC, Iversen L, Fielding S, Lidegaard Ø. Contemporary hormonal contraception and the risk of breast cancer. N Engl J Med. 2017;377(23):2228–39. 29211679 10.1056/NEJMoa1700732

[5] Claus EB, Risch N, Thompson WD. Autosomal dominant inheritance of early-onset breast cancer: implications for risk prediction. 49, Obstetrical and Gynecological Survey. 1994. pp. 401–2. 10.1002/1097-0142(19940201)73:3<643::aid-cncr2820730323>3.0.co;2-5 8299086

[6] Peto J, Mack TM. High constant incidence in twins and other relatives of women with breast cancer. Nat Genet. 2000;26:411–4. 11101836 10.1038/82533

[7] Economopoulou P, Dimitriadis G, Psyrri A, Beyond BRCA. New hereditary breast cancer susceptibility genes. Cancer Treat Rev. 2015;41:1–8. 25467110 10.1016/j.ctrv.2014.10.008

[8] Moorman PG, Havrilesky LJ, Gierisch JM, Coeytaux RR, Lowery WJ, Urrutia RP, et al. Oral contraceptives and risk of ovarian cancer and breast cancer among high-risk women: a systematic review and meta-analysis. J Clin Oncol. 2013;31:4188–98. 24145348 10.1200/JCO.2013.48.9021

[9] Iodice S, Barile M, Rotmensz N, Feroce I, Bonanni B, Radice P, et al. Oral contraceptive use and breast or ovarian cancer risk in BRCA1/2 carriers: a meta-analysis. Eur J Cancer. 2010;46:2275–84. 20537530 10.1016/j.ejca.2010.04.018

[10] Brohet RM, Goldgar DE, Easton DF, Antoniou AC, Andrieu N, Chang-Claude J, et al. Oral contraceptives and breast cancer risk in the international BRCA1/2 carrier cohort study: a report from EMBRACE, GENEPSO, GEO-HEBON, and the IBCCS collaborating group. J Clin Oncol. 2007;25(25):3831–6. 17635951 10.1200/JCO.2007.11.1179

[11] Woodward ER, Van Veen EM, Gareth Evans D. From BRCA1 to polygenic risk scores: mutation-associated risks in breast cancer-related genes. Breast Care. 2021;16(3):202. 34248461 10.1159/000515319 PMC8248775

[12] Zhang H, Ahearn TU, Lecarpentier J, Barnes D, Beesley J, Qi G, et al. Genome-wide association study identifies 32 novel breast cancer susceptibility loci from overall and subtype-specific analyses. Nat Genet. 2020;52(6):572. 32424353 10.1038/s41588-020-0609-2 PMC7808397

[13] Pashayan N, Duffy SW, Chowdhury S, Dent T, Burton H, Neal DE, et al. Polygenic susceptibility to prostate and breast cancer: implications for personalised screening. Br J Cancer. 2011;104:1656–63. 21468051 10.1038/bjc.2011.118 PMC3093360

[14] Allen N, Sudlow C, Downey P, Peakman T, Danesh J, Elliott P, et al. UK biobank: current status and what it means for epidemiology. Health Policy Technol. 2012;1(3):123–6.

[15] Sudlow C, Gallacher J, Allen N, Beral V, Burton P, Danesh J, et al. UK biobank: an open access resource for identifying the causes of a wide range of complex diseases of middle and old age. PLoS Med. 2015;12(3):e1001779. 25826379 10.1371/journal.pmed.1001779 PMC4380465

[16] Allen NE, Lacey B, Lawlor DA, Pell JP, Gallacher J, Smeeth L, et al. Prospective study design and data analysis in UK biobank. Sci Transl Med. 2024;16(729):eadf4428. 38198570 10.1126/scitranslmed.adf4428 PMC11127744

[17] Thiébaut ACM, Bénichou J. Choice of time-scale in cox’s model analysis of epidemiologic cohort data: a simulation study. Stat Med. 2004;23(24):3803–20. 15580597 10.1002/sim.2098

[18] Tamim H, Tahami Monfared AA, LeLorier J. Application of lag-time into exposure definitions to control for protopathic bias. Pharmacoepidemiol Drug Saf. 2007;16:250–8. 17245804 10.1002/pds.1360

[19] Thompson DJ, Wells D, Selzam S, Peneva I, Moore R, Sharp K et al. UK biobank release and systematic evaluation of optimised polygenic risk scores for 53 diseases and quantitative traits. MedRxiv. 2022;2022.06.16.22276246.

[20] Thompson DJ, Wells D, Selzam S, Peneva I, Moore R, Sharp K et al. A systematic evaluation of the performance and properties of the UK biobank polygenic risk score (PRS) release. PLoS ONE. 2024;19(9). 10.1371/journal.pone.0307270 PMC11410272 39292644

[21] Horn J, Åsvold BO, Opdahl S, Tretli S, Vatten LJ. Reproductive factors and the risk of breast cancer in old age: A Norwegian cohort study. 139, Breast Cancer Research and Treatment. 2013. pp. 237–43. 10.1007/s10549-013-2531-0 23605085

[22] Kelsey JL, Gammon MD, John EM. Reproductive factors and breast cancer. Epidemiol Rev. 1993;15:36–47. 8405211 10.1093/oxfordjournals.epirev.a036115

[23] Friedenreich CM. Review of anthropometric factors and breast cancer risk. Eur J Cancer Prev. 2001;10:15–32. 11263588 10.1097/00008469-200102000-00003

[24] Downing A, Prakash K, Gilthorpe MS, Mikeljevic JS, Forman D. Socioeconomic background in relation to stage at diagnosis, treatment and survival in women with breast cancer. Br J Cancer. 2007;96:836–40. 17311024 10.1038/sj.bjc.6603622 PMC2360059

[25] Pakzad R, Nedjat S, Yaseri M, Salehiniya H, Mansournia N, Nazemipour M, et al. Effect of smoking on breast cancer by adjusting for smoking misclassification bias and confounders using a probabilistic bias analysis method. Clin Epidemiol. 2020;12:557–68. 32547245 10.2147/CLEP.S252025 PMC7266328

[26] Therneau TM, Grambsch PM. The Cox Model. Modeling survival data: extending the Cox Model\. New York, NY\: Springer\; 2000. pp. 39–77.

[27] Andersen PK, Gill RD. Cox’s Regression Model for Counting Processes: A Large Sample Study. Vol. 10, The Annals of Statistics. 2007. pp. 1100–1120\.

[28] Johansson T, Fowler P, Ek WE, Skalkidou A, Karlsson T, Johansson A. Oral Contraceptives, hormone replacement Therapy, and stroke risk. Stroke. 2022;53(10):3107–15. 35735009 10.1161/STROKEAHA.121.038659

[29] Zhou Z, Rahme E, Abrahamowicz M, Pilote L. Survival bias associated with time-to-treatment initiation in drug effectiveness evaluation: A comparison of methods. Am J Epidemiol. 2005;162(10):1016–23. 16192344 10.1093/aje/kwi307

[30] Karlsson T, Johansson T, Hoglund J, Ek WE, Johansson Å. Time-dependent effects of oral contraceptive use on breast, ovarian, and endometrial cancers. Cancer Res. 2021;81(4):1153–62. 33334812 10.1158/0008-5472.CAN-20-2476

[31] Iversen L, Sivasubramaniam S, Lee AJ, Fielding S, Hannaford PC. Lifetime cancer risk and combined oral contraceptives: the Royal college of general practitioners’ oral contraception study. Am J Obstet Gynecol. 2017;216(6):e5801–9. 10.1016/j.ajog.2017.02.002 28188769

[32] Mars N, Kerminen S, Tamlander M, Pirinen M, Jakkula E, Aaltonen K, et al. Comprehensive inherited risk Estimation for risk-Based breast cancer screening in women. J Clin Oncol. 2024;42(13):1477–87. 38422475 10.1200/JCO.23.00295 PMC11095905

[33] Baliakas P, Munters AR, Kämpe A, Tesi B, Bondeson ML, Ladenvall C, et al. Integrating a polygenic risk score into a clinical setting would impact risk predictions in Familial breast cancer. J Med Genet. 2024;61(2):150–4. 37580114 10.1136/jmg-2023-109311 PMC10850617

[34] Mavaddat N, Michailidou K, Dennis J, Lush M, Fachal L, Lee A, et al. Polygenic risk scores for prediction of breast cancer and breast cancer subtypes. Am J Hum Genet. 2019;104(1):21–34. 30554720 10.1016/j.ajhg.2018.11.002 PMC6323553

[35] Kamiza AB, Toure SM, Vujkovic M, Machipisa T, Soremekun OS, Kintu C, et al. Transferability of genetic risk scores in African populations. Nat Med 2022. 2022;28(6):6. 10.1038/s41591-022-01835-x PMC9205766 35654908

[36] Ivansson EL, Johansson T, Karlsson T, Johansson Å. Oral contraceptive use increases bone density and reduces the risk of osteoporosis. Eur J Epidemiol. 2025. 10.1007/s10654-025-01273-2 PMC12537589 40632378

[37] Endometrial cancer and oral contraceptives: an individual participant meta-analysis of 27 276 women with endometrial cancer from 36 epidemiological studies. Lancet Oncol. 2015;16(9):1061–70. 10.1016/S1470-2045(15)00212-0 26254030

[38] Iversen L, Fielding S, Lidegaard Ø, Mørch LS, Skovlund CW, Hannaford PC. Association between contemporary hormonal contraception and ovarian cancer in women of reproductive age in denmark: Prospective, nationwide cohort study. BMJ (Online). 2018;362:k3609. 30257920 10.1136/bmj.k3609 PMC6283376

