# Supplementary material for: Breast cancer risk during oral contraceptive use in women with high polygenic risk
Source: Breast Cancer Res. 2025 Dec 1;27:215. doi: 10.1186/s13058-025-02177-5 (PMC12690897; doi:10.1186/s13058-025-02177-5)
Supplement: Supplementary file 1 — Supplementary Material 1 [file 13058_2025_2177_MOESM1_ESM.docx]

**Supplementary Tables**

[**Supplementary Table S1**. **Identification of Breast Cancer cases in UK biobank**. 2](#_Toc208393462)

[**Supplementary Table S2. Variables identified in UK biobank.** 3](#_Toc208393463)

[**Supplementary Table S3. Hazard ratios of oral contraceptive use on breast cancer risk in the during oral contraceptive use model, including adjustment for smoking status.** 5](#_Toc208393464)

[**Supplementary Table S4. Effect of current oral contraceptive (OC) use and genetic predisposition on breast cancer risk, including adjustment for smoking status**. 5](#_Toc208393465)

**Supplementary Table S1**. **Identification of breast cancer cases in UK biobank**. *The age at first diagnosis was used in the analyses. If a participant has several ages reported in the data-sources, the lowest age was used regardless of the source of the data.*

| **Cancer diagnoses** | | | | | **Age first diagnosed** |
| --- | --- | --- | --- | --- | --- |
| **Data source** | **Type** | **Data-fields*** | **Coding** | **Code** | **Data-fields*** |
| Hospital inpatient | Register data | 41270 | ICD-10 | C50 | 41280 |
| Hospital inpatient | Register data | 41271 | ICD-9 | 174 | 41281 |
| Cause of death | Register data | 40001/40002 | ICD-10 | C50 | 40007 |
| Cancer type | Register data | 40006 | ICD-10 | C50 | 40008 |
| Cancer type | Register data | 40013 | ICD-9 | 174 | 40008 |

*Data-fields refers to the identifier in the UK Biobank database.

#### **Supplementary Table S2. Variables identified in UK Biobank.**

| **Variables** | **UKB Data-fields** | **Data source** |
| --- | --- | --- |
| **Exposure variables^1^** |  |  |
| Ever taken oral contraceptives (OC) | 2784 | Self-reported |
| Age started OC | 2794 | Self-reported |
| Age stopped OC | 2804 | Self-reported |
| Medication | 6153 | Self-reported |
| **Fixed covariates** |  |  |
| Age^2^ | 21003 | Registry |
| Year of birth^2^ | 34 | Registry |
| Body mass index (BMI) ^3^ | 21001 | Measured |
| Townsend deprivation index (TDI) ^2^ | 189 | Derived variable |
| Age at menarche ^3^ | 2714 | Self-reported |
| Number of live births^3^ | 2734 | Self-reported |
| Polygenic risk score (PRS)^5^ | 26220 | Derived variable |
| **Time varying covariates** |  |  |
| Menopausal status (yes/no)^5^ | 2724 | Self-reported |
| Age at menopause^5^ | 3581, 2824, 21003 | Self-reported |
| Ever had hysterectomy^5^ | 3591 | Self-reported |
| Age at hysterectomy^5^ | 2824 | Self-reported |
| Ever used hormone replacement therapy (HRT)^6^ | 2814 | Self-reported |
| Age started HRT^6^ | 3536 | Self-reported |
| Smoking Status^7^ | 20116 | Self-reported |
| Age started smoking (current smokers) | 3436 | Self-reported |
| Age started smoking (former smokers) | 2867 | Self-reported |
| Age stopped smoking (former smokers) | 2897 | Self-reported |

*1. Participants who responded: "do not know," "prefer not to answer," or did not provide an answer to the question "Have you ever taken the contraceptive pill?" were excluded from the study. For those who answered "yes," additional data on the "age when started oral contraceptive pill" were extracted from the touchscreen questionnaire. Participants who responded, "do not know," "prefer not to answer," or did not provide an answer regarding their age at initiation of oral contraceptive (OC) use were also excluded. Furthermore, cross-checks were performed to identify inconsistencies, such as individuals reporting current use of "oral contraceptive pill or minipill" as a response to the question “Do you regularly take any of the following medications?” but not being classified as ever-users, or participants categorized as never-users but reporting an age at OC initiation. No such discrepancies were found, and thus no additional exclusions were necessary. Participants who indicated that they were previous users, but did not report age at discontinuation, "age stopped OC use", were excluded (these individuals responded with "do not know" or "prefer not to answer". Participants who were still using OC at the time of the initial assessment (the end of follow-up), were considered current users until the end of follow-up.*

*2. Demographic and socioeconomic data, including birth year and Townsend deprivation index (TDI), were obtained from the National Health Service Primary Care Trust register before enrolment in UKB.*

*3. Body mass index (BMI) was calculated using height and weight measurements taken at the initial assessment visit. Age at menarche (onset of menstruation) and the number of live births were obtained from the touchscreen questionnaire.*

*4. The polygenic risk scores (PRS) for breast cancer (breast cancer) used in this study were precomputed by Genomics PLC (Thompson et al. 2022) under UKB project 9659 and provided through UKB (data field 26220- Standard PRS for breast cancer). PRS scores were generated using a Bayesian approach applied to meta-analyzed GWAS summary statistics, derived entirely from external GWAS data (not containing UK Biobank). Ιndividual PRS values were computed as the genome-wide sum of the per-variant posterior effect size multiplied by allele dosage, providing a comprehensive assessment of genetic risk. Reference. Thompson, D. J., Wells, D., Selzam, S., Peneva, I., Moore, R., Sharp, K., ... & Weale, M. E. (2022). UK Biobank release and systematic evaluation of optimised polygenic risk scores for 53 diseases and quantitative traits. MedRxiv, 2022-06. doi: 10.1101/2022.06.16.22276246.*

*5. Menopausal status was determined based on responses from the initial assessment. Participants were asked, "Have you had your menopause (periods stopped)?" with response options including "yes," "no," "not sure – had a hysterectomy," "not sure – other reason," and "prefer not to answer." Those who selected "prefer not to answer" or did not respond were excluded from all analyses. Women who answered "no" were classified as premenopausal, while those selecting "yes" or either of the "not sure" categories were grouped as menopausal. Age at menopause was derived from responses provided in the touchscreen questionnaire at the initial assessment visit. This information was collected from the reported age at menopause (for those answering "yes"), age at hysterectomy (for those uncertain due to a hysterectomy), or age at assessment (for participants uncertain due to other reasons). Additionally, all participants, except those who had already indicated "not sure – had hysterectomy," were asked whether they had undergone a hysterectomy (removal of the uterus). Those responding, "not sure," "prefer not to answer," or who did not provide an answer were excluded. Participants who did not specify their age at menopause or hysterectomy were also excluded. Further checks were performed to identify discrepancies, such as individuals indicating "no" for menopause but providing an age at menopause, or those uncertain about their menopausal status due to hysterectomy but not reporting a hysterectomy. No such cases were identified, and no additional exclusions were required. Both menopause and hysterectomy were modelled as dichotomous (yes/no), time-varying variables.*

*6. Regarding Hormone Replacement Therapy (HRT), women who responded, "do not know," "prefer not to answer," or did not provide an answer to the question "Have you ever used hormone replacement therapy?" were excluded from all analyses. For participants who answered "yes," follow-up data regarding "age when started HRT" were extracted. Those who did not provide this information or responded "do not know" or "prefer not to answer" were excluded. Additionally, checks were conducted to identify inconsistencies where participants classified as never-HRT users had an age at HRT initiation. No such inconsistencies were found, and thus no further exclusions were necessary.*

*7. Smoking status was assessed using the touchscreen questionnaire, categorizing participants as never, previous, or current smokers. Those selecting "prefer not to answer" to the question regarding their smoking status were excluded from all analyses including smoking status. Previous and current smokers were further asked to provide their "age started smoking," with current smokers also providing "age stopped smoking." Participants who answered "do not know" or "prefer not to answer" for these questions were excluded from analyses involving smoking status. Additionally, data validation checks ensured that no participants classified as never-smokers had reported an age at smoking initiation. Participants with missing data for smoking initiation or discontinuation who were not categorized as never-smokers were excluded from all smoking-related analyses.*

**Supplementary Table S3. Hazard ratios of oral contraceptive use on breast cancer risk in the during oral contraceptive use model, including adjustment for smoking status.** *Analysed without polygenic risk score (PRS), with PRS as a continuous fixed covariate, and with PRS as an interaction factor. (N: Number of participants, n: number of observations, events: number of breast cancer cases). Never users were used as the reference group in all analyses.*

| **Exposure** | **Without PRS** | | **With PRS** | | **With PRS*OC interaction term** | |
| --- | --- | --- | --- | --- | --- | --- |
| **Participants** | 163,707 | | 158,448 | | 158,448 | |
| **Breast cancer** | 1,739 | | 1,605 | | 1,605 | |
|  | **HR (95% CI)** | **P-value** | **HR (95% CI)** | **P-value** | **HR (95% CI)** | **P-value** |
| **During OC use** | 1.20 (1.03,1.40) | 0.018 | 1.22 (1.05,1.43) | 0.010 | 1.26  7 (1.08,1.49) | 0.0044 |
| **PRS for breast cancer** |  |  | 1.84 (1.75,1.93) | <2e-16 | 1.87 (1.77,1.98) | <2e-16 |
| **OC*PRS (interaction term)** |  |  | - |  | 0.92 (0.82,1.04) | 0.21 |

**Supplementary Table S4. Effect of current oral contraceptive (OC) use and genetic predisposition on breast cancer risk, including adjustment for smoking status**. Participants were categorized into low, medium, and high genetic risk groups based on tertiles of the polygenic risk score (PRS). The reference group consisted of never users of OC with medium genetic risk. A total of 158,448 women were included in the analysis, with 1,966 breast cancer events observed during follow-up.

|  | **HR (95% CI), P-value** |
| --- | --- |
| Never OC, Low Risk | 0.42 (0.35–0.50), p < 2e-16 |
| During OC, Low Risk | 0.65 (0.48–0.88), p = 0.005 |
| During OC, Medium Risk | 1.21 (0.95–1.55), p = 0.11 |
| Never OC, High Risk | 1.97 (1.74–2.24), p < 2e-16 |
| During OC, High Risk | 2.27 (1.86–2.78), p = 1.7e-15 |
